# Improvement of *Lotus japonicus* hairy root induction and development of a mycorrhizal symbiosis system

**DOI:** 10.1002/aps3.1141

**Published:** 2018-05-07

**Authors:** Yunjian Xu, Fang Liu, Guomin Han, Wei Wang, Suwen Zhu, Xiaoyu Li

**Affiliations:** ^1^ Key Laboratory of Crop Biology of Anhui Province Anhui Agricultural University Hefei 230036 China; ^2^ School of Life Sciences Anhui Agricultural University Hefei 230036 China; ^3^ College of Agronomy Anhui Agricultural University Hefei 230036 China

**Keywords:** *Agrobacterium rhizogenes*, hairy root, *Lotus japonicus*, *Rhizophagus irregularis*

## Abstract

**Premise of the Study:**

We describe a highly efficient in vitro *Lotus japonicus* hairy root transformation system that is useful for the investigation of mycorrhizal symbiosis.

**Methods and Results:**

The *Agrobacterium rhizogenes*–mediated transformation method was improved based on the binary plasmid (pCAMBIA *1304*) harboring green fluorescent protein and β‐glucuronidase genes for rapid detection. Transgenic hairy roots were grown within 13 days. These in vitro–cultured hairy roots can be inoculated with *Rhizophagus irregularis*, thus facilitating the investigation of the symbiosis between *L. japonicus* and arbuscular mycorrhizal fungi.

**Conclusions:**

Compared with existing techniques, our protocol provides a simple and efficient *A. rhizogenes–*mediated transformation system for *L. japonicus*. The rapid induction of hairy roots can shorten the experimental time by at least one week.

As a model legume plant, *Lotus japonicus* (Regel) K. Larsen has been used to investigate the plant–rhizobial and the plant–arbuscular mycorrhizal (AM) fungal relationships that lead to the fixation of nitrogen and the increased uptake of phosphorus, respectively (Sato et al., 2008; Clemow et al., 2011). Efficient hypocotyl transformation regeneration of *L. japonicus* using *Agrobacterium tumefaciens* has been described previously (Stiller et al., 1997; Sasaki et al., 2013). *Agrobacterium rhizogenes*–mediated transformation in legumes was first described in *L. corniculatus* L. (Jensen et al., 1986; Petit et al., 1987). Such transgenic plants (transformed roots with a non‐transformed plant) have been nodulated directly (Hansen et al., 1989), but the current approach is not suitable for *L. japonicus*. Because many non‐transformed roots may grow from the stem of *Lotus* L., a simple and rapid approach is needed to stably integrate transgene vectors and make the target functionally expressed.

For decades, increasing attention has been paid to investigating the symbiotic relationship between plants and AM fungi. Although it is well known that AM fungi can enhance plant resistance to biotic and abiotic stresses and improve plant nutrient uptake, the underlying molecular mechanisms are not yet fully understood. Given that the first connection between *L. japonicus* and AM fungi occurs in the plant root, it is important to study the interaction of AM fungi and plant roots in a sterile environment. In this study, we developed a fast and efficient method for *A. rhizogenes*–mediated transformation of *L. japonicus* (ecotype MG20) root. The transgenic hairy roots of *L. japonicus* were reproduced for the study of symbiotic mycorrhizal root colonization.

In brief, our protocol includes five steps: (1) seed surface sterilization and germination, (2) transformation, (3) hairy root emergence, (4) hairy root selection, and (5) *Rhizophagus irregularis* (a well‐known species of AM fungi) inoculation of transformed hairy roots.

## METHODS AND RESULTS

### Sterilization and germination of seeds

The seeds of *L. japonicus* ecotype MG20 were used in this study (Okamoto et al., 2013). Seeds were sterilized with 12% (v/v) commercial KAO bleach (Kao Industrial Co. Ltd., Hong Kong, China) containing 0.1% (v/v) Tween 20 for 15 min. Disinfectant was removed and the foam was removed three times with 75% (v/v) alcohol; the seeds were then washed six times for 15 min with sterile water. Under sterile conditions, surface‐sterilized *L. japonicus* seeds were placed onto 1.2% agar in a Petri dish. The dishes were placed in the dark at 23°C overnight and then inverted for 24 h. After 48 h, seeds grew 1‐cm‐long roots with folded cotyledons. Compared to 2% sodium hypochlorite, KAO bleach and Tween‐mixed disinfectant are effective, with a higher seed germination rate (Table 1).

**Table 1:** The germination rate of *Lotus japonicus* ecotype MG20 seeds sterilized by KAO bleach and sodium hypochlorite solution without sandpaper scarification

Seed sterilization method	No. of germinated seeds	Total no. of seeds	Germination rate (%)
12% KAO bleach	198	270	73 ± 3.2
2% Sodium hypochlorite	170	306	55 ± 5.2

### Induction of hairy roots of *L. japonicus*

The *A. rhizogenes* strain LBA9402 was used in this study (Le Flem‐Bonhomme et al., 2004). The binary vector pCAMBIA1304 (BioVector NTCC) carrying a green fluorescent protein (GFP) gene and a β‐glucuronidase (GUS) gene was introduced into LBA9402 strains by the electroporation method (1800 V/cm, 9 msec). Transformants were selected in the presence of kanamycin and rifampicin, each at a final concentration of 50 mg/L, and positive‐transformant colonies were cultured in 5 mL of yeast mannitol broth medium (K_2_HPO_4_ 0.5 g/L, MgSO_4_·7H_2_O 0.2 g/L, NaCl 0.1 g/L, yeast extract 0.4 g/L, and D‐mannitol 10 g/L) by shaking at 28°C and 200 rpm for 48 h. Thereafter, transformant *A. rhizogenes* were applied to a solid yeast mannitol broth medium containing 50 mg/L kanamycin and 50 mg/mL rifampicin cultured in the dark at 28°C for 48 h.

**Figure 1:**
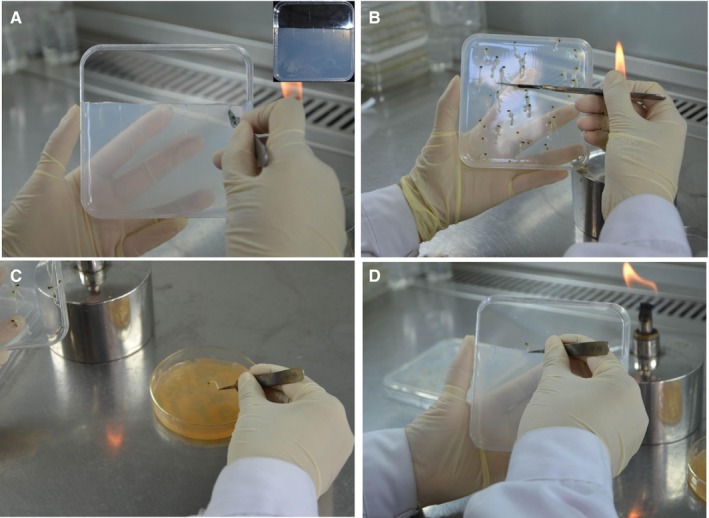
The procedure of inducing hairy roots from *Lotus japonicus*. (A) Remove part of the medium and then cut a number of vertical parallel incisions. (B) Section out the seedling radicles approximately 2 mm from the root tip with a sterile scalpel. (C) Coat the cut surface of the radicles with *Agrobacterium rhizogenes* for infection. (D) Place *A. rhizogenes*–coated seedlings into incisions in the medium.

Broughton and Dilworth (B&D) medium (1.0 × 10^−3^ M CaCl_2_·2H_2_O, 5.0 × 10^−4^ M KH_2_PO_4_, 1.0 × 10^−5^ M ferric citrate, 2.5 × 10^−4^ M MgSO_4_·7H_2_O, 1.5 × 10^−3^ M K_2_SO_4_, 1.0 × 10^−6^ M MnSO_4_·7H_2_O, 2.0 × 10^−6^ M H_3_BO_3_, 2.0 × 10^−7^ M CuSO_4_·5H_2_O, 1.0 × 10^−7^ M CoSO_4_·7H_2_O, 1.0 × 10^−7^ M Na_2_MoO_4_·2H_2_O, 5.0 × 10^−7^ M ZnSO_4_·H_2_O, and 1.0 × 10^−3^ M KNO_3_; Broughton and Dilworth, 1971) with 1.2% agar added to 150 μM acetosyringone (acetosyringone can induce *Agrobacterium vir* gene activation, increasing the induction rate) was used as a co‐cultivation medium for hairy root growth. B&D medium in square dishes (12 × 12 cm) was reduced by one‐third, and a number of vertical parallel incisions were cut into the surface of the medium (Fig. 1A). Two days after germination, the seedling radicles were sectioned out approximately 2 mm from the root tip with a sterile scalpel (Fig. 1B). The cut surface of the radicles was coated with *A. rhizogenes* for infection (Fig. 1C) and the inoculated roots of the seedlings were then placed in the incisions of the B&D medium, with the cotyledons outside (Fig. 1D). Finally, the dishes were sealed with Parafilm and medical microporous tape (3M, Maplewood, Minnesota, USA) and placed in a growth chamber at 28°C overnight to increase transformation efficiency. The dishes were then transferred vertically to a 23°C growth chamber (16‐h photoperiod) for 10 days. Unlike previous studies (Appendix 1), hairy root induction and elongation in this study were all performed with B&D medium rather than transferred to a different root elongation medium.

### Hairy root selection and subculture

**Figure 2:**
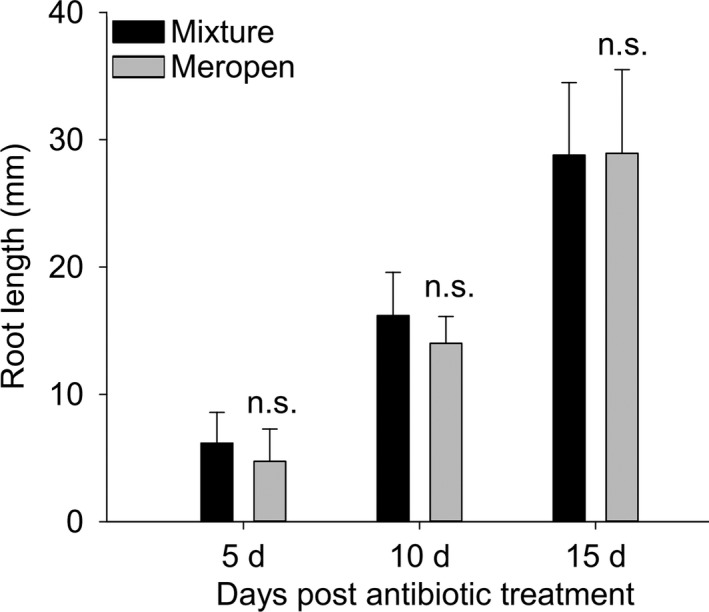
The root length of transgenic roots treated by a mixture of cephalosporin and timentin compared to meropen after 5, 10, and 15 days. Error bar represents SD with 20 biological replications.

The elongated shoot of *L. japonicus* from the co‐cultivation medium was cut so that only hairy roots remained. The hairy roots were first washed in sterile water and then washed with a solution containing 300 mg/L cephalosporin and 200 mg/L timentin for 5 min, followed by soaking for 15 min. Next, sterile water was used to remove the surface antibiotics from the hairy roots, which were then dried using filter paper and placed onto the surface of the selection medium and cultured at 23°C. Hairy roots were then cultured on a modified Strullu–Romand medium with 0.3% Phytagel (Sigma, St. Louis, Missouri, USA; Li et al., 2013) containing 100 mg/L cephalosporin, 100 mg/L timentin, and 50 mg/L hygromycin for one week to eliminate *A. rhizogenes* contamination and to select transgenic hairy roots. We compared the root growth rate with the cephalosporin and timentin mixture to meropen (12.5 μg/mL), which was used to eliminate *A. rhizogenes* AGL1 in the *L. japonicus* stable transformation, and found no significant difference (Fig. 2).

### Detection of transgenic roots

**Figure 3:**
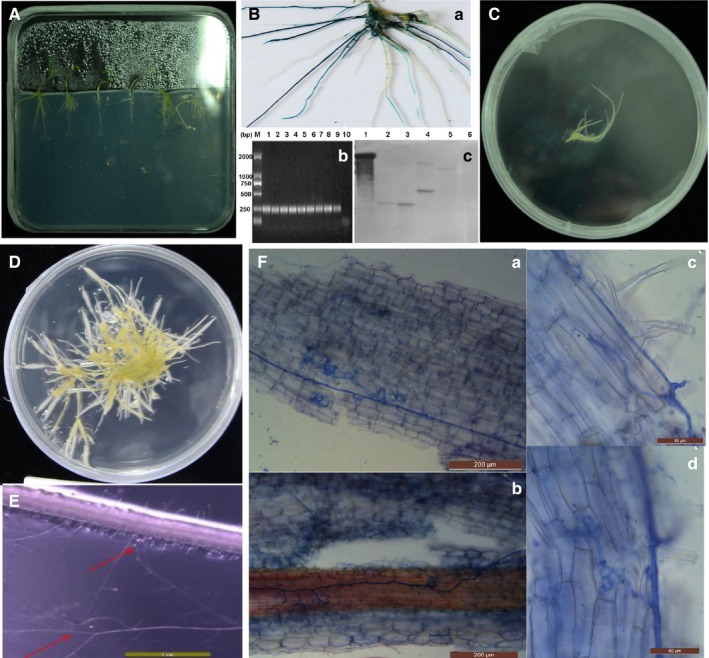
Acquisition of hairy roots from *Lotus japonicus* and the establishment of symbiosis with *Rhizophagus irregularis*. (A) Growth of hairy roots from *L. japonicus*. (B) X‐Gluc staining (a), PCR (b), and Southern blot (c) identification of transgenic hairy roots. (b) M represents the 2000‐bp marker, line 1 represents pCAMBIA *1304*, lines 2 through 9 represent transgenic hairy roots, and line 10 represents non‐transgenic roots. The target band is located at 250 bp. (c) Line 1 represents pCAMBIA *1304* plasmid DNA digested with *Eco*R1; lines 2 through 5 represent transgenic hairy roots lines; line 6 represents non‐transgenic roots. (C) Hairy roots have been transferred to modified Strullu–Romand medium. (D) Transgenic hairy root growth. (E) *Rhizophagus irregularis* inoculation with transgenic hairy root growth. Red arrows indicate fungal hyphae. (F) The appearance of trypan blue–stained roots of arbuscular mycorrhizal–colonized transgenic hairy roots two weeks after inoculation. (a) and (b) show fungal hyphae, (c) shows fungal entry points, and (d) shows fungal arbuscules.

After 10 days of vertical growth, a tumor appeared on the wound and hairy roots clearly initiated from the tumor site after infection (Fig. 3A). The GUS reporter was used to identify the transformed hairy roots and to quantify the efficiency of the transformation. GUS staining, as well as PCR, was used for the confirmation of positive transformants. For GUS histochemical staining, hairy roots were incubated with X‐Gluc (Sangon Biotech, Shanghai, China) in the dark at 37°C for 24 h. GUS staining indicated GUS‐positive hairy roots (Fig. 3B). The *gus* gene was further amplified via PCR using DNA from hairy roots to verify the positive transgenic hairy roots (Fig. 3B). The genomic DNA of the transgenic hairy root was extracted by the cetyltrimethylammonium bromide method (Clarke, 2009). The forward and reverse primers were: 5ʹ‐GGGCAACAAGCCGAAAGA‐3ʹ and 5ʹ‐GGTGTGAGCGTCGCAGAAC‐3ʹ. The PCR products were expected to be approximately 259 bp. The reaction conditions were: 32 cycles of 95°C for 10 s, 55°C for 30 s, and 72°C for 30 s. For the negative control, the DNA of non‐transgenic MG20 was added to the reaction mix. PCR results further verified the positive transgenic hairy roots (Fig. 3B). To confirm that the transgenes were integrated into the MG20 hairy root genome, Southern blot analysis was carried out. Hairy root genomic DNA was digested with *Eco*R1 (Promega Corporation, Madison, Wisconsin, USA) and probed with the *Hyg* gene. As shown in Fig. 3B, all the tested transgenic hairy roots were carrying one to two copies of the transgene.

### Colonization of *L. japonicus* hairy roots by *R. irregularis*

Excised decontaminated transgenic hairy roots were subcultured onto modified Strullu–Romand medium in the absence of antibiotics. After the transgenic roots of *L. japonicus* were obtained (Fig. 3C), a symbiotic relationship between hairy roots and *R. irregularis* was further established (Fig. 3D, E). After one week, approximately 100 fungal spores of *R. irregularis* produced from carrot hairy root co‐culture (Chabot et al., 1992) were placed on the surface of *L. japonicus* hairy roots (Fig. 3D). After an additional week, hairy roots were colonized by the fungi (Fig. 3E, F). *Rhizophagus irregularis*–colonized hairy roots were detected by trypan blue staining, which can stain the arbuscule structure and intracellular hyphae. Trypan blue staining was performed as previously described: treatment with 10% KOH, heating at 90°C for 1 h, treatment with 2% HCl solution for 5 min, staining with trypan blue, and then the roots were transferred into lactic acid and glycerin for destaining (Liu et al., 2016). The dyed roots were then examined beneath a microscope. The fungal arbuscule structures in the hairy roots were clearly detected by trypan blue staining (Fig. 3F), and the structure of fungal hyphae, fungal arbuscules, and fungal entry points in the transgenic roots were distinctly visible under a light microscope after *R. irregularis* colonization (Fig. 3F).

## CONCLUSIONS

Compared with previously reported methods, this protocol has the following five advantages. First, seeds sterilized by KAO bleach and Tween‐mixed disinfectant were effective, with a higher germination rate compared to sterilization with the 2% sodium hypochlorite solution. Second, this protocol used only two types of simple medium (1.2% agar and B&D medium) from seed germination to hairy root induction. Third, the optimized sterilization step can remove as much of the *Agrobacterium*‐coated hairy roots as possible with less damage to the hairy roots. Fourth, the time from germination to hairy root induction was 13 days, which saves a minimum of one week of experimental time. Finally, roots cultured in vitro are useful for symbiosis‐related research.

## Appendix 1 A comparison of the different methods of obtaining hairy roots from *Lotus japonicus*.

**Table:**

Medium	Time	Reference
1% agar, B5 standard medium, callus‐inducing medium, shoot‐inducing medium, shoot elongation medium, root‐inducing medium, root elongation medium	4 months	Lombari et al., 2003
Jensen medium, co‐cultivation medium, hairy roots emergence medium	53 days	Stougaard et al., 2005
1% agar, co‐cultivation medium, hairy root elongation medium	20–24 days	Okamoto et al., 2013
1.2% agar, B&D medium	13 days	This study
